# Case-Control Study of Rotavirus Vaccine Effectiveness Compared to Test-Negative Controls or Hospital Controls

**DOI:** 10.2188/jea.JE20180054

**Published:** 2019-08-05

**Authors:** Kaoru Araki, Megumi Hara, Chisato Shimanoe, Yuichiro Nishida, Muneaki Matsuo, Keitaro Tanaka

**Affiliations:** 1 Department of Preventive Medicine, Faculty of Medicine, Saga University, Saga, Japan; 2 Department of Pediatrics, Faculty of Medicine, Saga University, Saga, Japan

**Keywords:** hospital controls, rotavirus, vaccine effectiveness, test-negative controls

## Abstract

**Background:**

Selection of test-negative controls takes less time and costs less than traditional control selection for evaluating vaccine effectiveness (VE). Here, rotavirus VE was evaluated using hospital controls and compared with test-negative controls to determine whether using the latter can substitute for the former.

**Methods:**

We recorded gastroenteritis in children from 2 months to 2 years of age at six medical facilities in Saga City between January 4th and May 31st, 2014. Stools from all identified acute gastroenteritis patients were tested for rotavirus using immunochromatography. Rotavirus gastroenteritis (RVGE) cases had test-positive stool, whereas test-negative controls had gastroenteritis but no rotavirus infection; hospital controls were outpatients visiting the same facility for indications other than gastroenteritis. Vaccination status was verified by inspecting maternal and child health records, and demographic data were obtained from a questionnaire completed by the patients’ guardians or from the medical records. Unconditional logistic regression analysis was used to adjust for possible confounding factors.

**Results:**

Sixty-four RVGE cases, 260 test-negative controls, and 589 hospital controls were enrolled. The characteristics of the two control groups, including RV vaccination history, were similar. The RVGE cases were more likely to have used daycare services than children from either of the two control groups. The VE against RVGE estimated using hospital controls was 86.6% (95% confidence interval [CI], 55.9–96.0%), very similar to the VE using test-negative controls (84.9% [95% CI, 49.6–95.5%]).

**Conclusions:**

The estimated VE using test-negative controls and hospital controls is similar. Therefore, test-negative controls are considered appropriate for establishing VE.

## INTRODUCTION

Rotavirus vaccines were introduced in 2006 in the form of two live oral vaccines, a monovalent human rotavirus vaccine (RV1, Rotarix^®^, GlaxoSmithKline Biologicals, Rixansart, Belgium) and a pentavalent bovine-human reassortant vaccine (RV5, RotaTeq^®^, Merck & Co., Inc., Rahway, NJ, USA) which have been licensed in >100 countries.^1^^,^^2^ In Japan, RV1 and RV5 became available on the private market in November 2011 and July 2012, respectively, following establishment of efficacy in randomized controlled trials (RCTs).^3^^,^^4^ According to these RCTs, the efficacy of RV1 against all grades of rotavirus gastroenteritis (RVGE) and against severe RVGE was 79.3% (95% confidence interval [CI], 60.5–89.8%) and 91.6% (95% CI, 62.4–99.1%), respectively^3^; for RV5, respective values were 74.5% (95% CI, 39.9–90.6%) and 80.2% (95% CI, 62.4–99.1%).^4^ Vaccine efficacy is evaluated in an RCT, where subjects are usually selected from healthy people then randomly allocated to two groups; one group is given a real vaccine and the other is given a placebo. They are followed up carefully for adverse events and disease incidence. In real situations, on the other hand, vaccinated people are not necessarily healthy and they decide to be vaccinated by their own choice. Thus, the efficacy as determined in an RCT does not reflect the real situation, and the generalizability of efficacy data from these trials is limited. In contrast, vaccine effectiveness (VE), which is evaluated in real-world situations, is more useful for practical application. Because effectiveness is affected by vaccine-related factors, such as immunogenicity, and also non-vaccine related factors, such as host factors and outcome definitions, the effectiveness may be lower than efficacy.^5^ Hence, determining VE after general introduction of the product is important.^6^

Case-control studies are commonly used for evaluation of VEs because they yield results rapidly from a moderate sample size.^7^ The most difficult part of a case-control study is often the selection of the appropriate controls. The main issue in this respect in rotavirus vaccine studies is that exposure to the virus among the controls should be the same as in the cases. Traditionally, controls are recruited from the community where the cases reside, or from hospitals that the cases also visit. However, possible confounding by healthcare-seeking behaviors needs to be considered in case-control studies when using traditional controls. In general, the probability of visiting a hospital, which is influenced by healthcare-seeking behavior, is greater in vaccinated than in unvaccinated individuals. Thus, the likelihood of there being more vaccinated individuals among cases recruited from hospitals tends to be greater than from the general population. In contrast, when controls are selected from residents without symptoms who have not visited a hospital, the likelihood of having been vaccinated is not affected by healthcare-seeking attitudes. As a result, the odds ratio of cases and community controls tends to increase, and VE may be underestimated. However, when controls are selected from hospital patients, the probability of having visited the hospital is greater in vaccinated than in unvaccinated patients but might differ according to how ill the patients are. Thus, the direction of potential bias cannot be anticipated.

In the last decade, a design employing test-negative controls has on occasion been adopted to evaluate VE for acute infectious diseases, such as influenza,^8^^–^^10^ rotavirus,^11^^,^^12^ and cholera.^13^^,^^14^ The pivotal advantage of this design is to cancel out bias due to healthcare-seeking behaviors between cases and controls. In a test-negative design, cases and controls can be selected from (the same group of) patients who visit clinics due to gastrointestinal symptoms. Hence, cases and test-negative controls have similar healthcare-seeking behavior for, in the present instance, gastroenteritis. Thus, using test-negative controls might reduce any confounding bias associated with gastroenteritis-associated health-seeking behavior relative to traditional controls. Furthermore, this form of control selection has the additional advantage of saving time and costs relative to traditional control selection. On the other hand, the VE evaluated with test-negative controls would depend on the sensitivity and specificity of the virus detection test employed. However, this is not a matter of concern for rotavirus VE because the sensitivity and specificity of enzyme immunoassays used for rotavirus identification is high.

Therefore, the objective of this study was to evaluate the effectiveness of rotavirus vaccine using both types of controls (hospital controls and test-negative controls), and to determine the suitability of using test-negative controls for establishing rotavirus VE.

## METHODS

### Study setting and design

The study was conducted in Saga City between January 4th and May 31st, 2014, a time of year corresponding to the rotavirus epidemic season.^15^ We conducted a case-control study using two control groups at six medical facilities (4 clinics and 2 hospitals); these were pediatric outpatient departments open during weekdays. One control group consisted of test-negative controls recruited through active surveillance for acute gastroenteritis, and the other used hospital controls. The survey protocol was approved by the Ethics Committee of Saga University Faculty of Medicine (NO. 25-41).

### Participants and definitions

All eligible participants were children between 2 months and 2 years of age, visiting medical facilities because of acute gastroenteritis (cases and test-negative controls) or because of other symptoms (hospital controls). Their guardians consented to this study. Acute gastroenteritis was defined as diarrhea (2 or more liquid stools or frequent stools) or vomiting (excluding coughing with vomiting). Children were excluded if symptom onset occurred within 14 days of rotavirus vaccination, or they were known to be infected with rotavirus before presentation. Written consent to participate was obtained from their guardians. Stool samples were collected from all acute gastroenteritis patients and tested for rotavirus at each facility using the same routine immunochromatographic assay (ICA; ImmunoCard^®^ SD Rota/Adeno, Standard Diagnostics, Inc., Geonggi-do, Korea). Even when the initial symptom was solely vomiting, without diarrhea, rectal swabs were tested for rotavirus.

A rotavirus case was defined as acute gastroenteritis testing positive for rotavirus, and a test-negative control as an individual with the same symptoms as the case but negative for rotavirus. A hospital control was defined as an individual from the same hospital where the case was enrolled, but having a condition unrelated to gastroenteritis. We estimated that a total of 56 RVGE cases would be needed to detect at least 80% VE against RVGE, using vaccination coverage of 40%.^16^

Because rotavirus vaccine is not expected to provide protection against non-rotavirus-related gastroenteritis, we anticipated no associations between vaccination and non-rotavirus-related gastroenteritis. To assess that, we compared non-rotavirus-related gastroenteritis and hospital controls.

### Information collection

We asked each child’s guardian to complete a self-administered questionnaire in order to collect the following information: sex, date of birth, birth weight, breastfed or not, receipt of daycare services, number of family members, number of siblings, parent age(s), underlying illnesses (food allergy, asthma, atopic dermatitis, epilepsy, otolaryngological disease, digestive organ disease, heart disease, Kawasaki disease, febrile convulsions, immunodeficiency, and congenital deformity), history of RVGE, history of rotavirus vaccination (and, if so, the number of doses, date of the last dose and the type of vaccine), and clinical symptoms and their date of onset (diarrhea, vomiting, fever, and seizure). In Japan, vaccination history is usually recorded in a mother-and-child health handbook maintained by the individuals concerned. Thus, information collected on vaccination status was verified using this record. When missing answers or inconsistent data were noted, accurate data were obtained via telephone interview with the subjects’ guardians.

### Severity classification

To assess the severity of disease in the outpatient setting, we adopted three of seven variables from a modified Vesikari score, MVS^17^ (a severity score): (1) maximal number of diarrheal stools per 24 h period (0 points: none, 1 point: 1–3, 2 points: 4–5, 3 points: ≥6), (2) maximal number of vomiting episodes per 24 h period (0 points: none, 1 point: 1, 2 points: 2–4, 3 points: ≥5), and (3) maximal fever (0 points: <37.0°C, 1 point: 37.1–38.4°C, 2 points: 38.5–38.9°C, 3 points: ≥39.0°C). The symptoms of all enrolled patients were scored. Because the scores in the top 10% and 25% of all patients were ≥7 and ≥5, respectively, we defined the severity of disease according to the following severity score: 1–4 was considered mild, 5–6 was considered moderate, and 7–9 was considered severe.

### Statistical analysis

We first performed bivariate analyses to assess differences in indicators of the clinical symptoms, and treatment between cases and test-negative controls, as well as background characteristics between cases, test-negative controls, and hospital controls using the chi-square test or Wilcoxon rank sum test. Background characteristic variables that exhibited a *P*-value <0.05 or appeared to be medically related to the disease were considered potential confounders requiring adjustment. Because we did not match for age and sex of individuals between cases and hospital controls or test-negative controls, unconditional logistic regression models were constructed to calculate the odds ratios (ORs) with 95% confidence intervals (CIs). We employed the following variables for adjustment: age (months), use of daycare (yes/no), and medical facility (in this analysis, we made this categorical variable act as a continuous variable by creating a dummy code because there were zeros in some strata). The VE against RVGE was estimated for at least one vaccine dose versus no dose, and was calculated as (1 − adjusted OR) × 100 (%). SAS statistical software (Ver. 9.3 for Windows; SAS Institute, Cary, NC, USA) was used for the analysis.

## RESULTS

**Figure 1.**
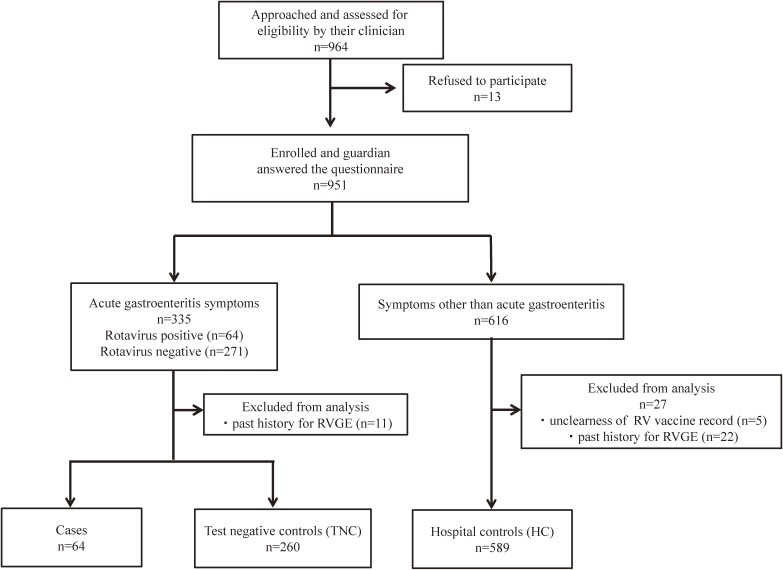
Flow chart of enrollment of rotavirus gastroenteritis cases, test-negative controls, and hospital controls. RVGE, rotarvirus gastroenteritis.

We approached a total of 964 patients’ guardians, 951 (98.7%) of whom consented to participate in this study and responded to the questionnaire. Among 335 acute gastritis patients, we excluded 11 (3.3%) who had a past history of RVGE. Hospital controls were 616 patients who visited the six medical facilities because of symptoms other than acute gastroenteritis. Of these, 5 (0.8%) whose vaccination history was not verified and 22 (3.6%) who had a past history of RVGE were excluded. Finally, 64 cases, 260 test-negative controls, and 589 hospital controls were enrolled in this study (Figure 1).

Table 1 shows the baseline characteristics of cases, hospital controls, and test-negative controls. The distribution of individuals attending the different medical facilities was significantly different for cases and hospital controls, as well as for test-negative controls and hospital controls. In comparison with test-negative controls and hospital controls, cases were significantly older and they were more likely to have used daycare services. The proportion of male patients was significantly higher in test-negative controls than in hospital controls. The proportions of vaccinated patients were significantly higher in test-negative controls and hospital controls than in the cases.

**Table 1.** Baseline characteristics of cases and controls

Variables	Cases, *n* = 64		TNC, *n* = 260		*P*-value^a^ comparing Cases and TNC	HC, *n* = 589		*P*-value^a^ comparing Cases and HC	*P*-value^a^ comparing TNC and HC
	
	Rotavirus-positive		Rotavirus-negative						
Demographics									
Age at onset, median months [range]	16	[3–22]	13	[3–23]	<0.01	13	[1–23]	<0.01	0.41
Sex: males, *n* (%)	34	(53.1)	146	(56.1)	0.66	283	(48.3)	0.46	0.03
Medical facilities									
A clinic	29	(45.3)	26	(33.1)	0.12	114	(19.4)	<0.01	<0.01
B clinic	9	(14.1)	58	(22.3)		156	(26.5)		
C clinic	16	(25.0)	54	(20.8)		145	(24.6)		
D clinic	1	(1.6)	23	(8.9)		53	(9.0)		
E hospital	7	(10.9)	24	(9.2)		41	(7.0)		
F hospital	2	(3.1)	15	(5.8)		80	(13.6)		

Additional history									
Underlying condition: Yes, *n* (%)	12	(18.7)	47	(18.1)	0.90	93	(15.8)	0.54	0.41
Premature (BW <2500 g)^b^, *n* (%)	59	(92.1)	229	(89.1)	0.47	536	(91.3)	0.81	0.31
Use of daycare service^c^: Yes, *n* (%)	38	(62.3)	104	(40.6)	<0.01	210	(35.8)	<0.01	0.18
Siblings: Yes, *n* (%)	42	(65.6)	155	(59.6)	0.38	352	(59.8)	0.36	0.96
Number of siblings, median [range]	2	[1–5]	2	[1–5]	0.79	2	[1–7]	0.99	0.65
Age of parents, median years [range]									
Mother^d^	31	[19–42]	32	[20–43]	0.31	32	[17–43]	0.26	0.94
Father^e^	33.1	[24–53]	33	[30–37]	0.62	33.5	[20–56]	0.64	0.89
Breastfed^f^: Yes, *n* (%)	30	(46.8)	141	(54.8)	0.25	321	(55.0)	0.21	0.96

Rotavirus vaccination status					<0.01			<0.01	0.11
Unvaccinated	61	(95.3)	178	(68.5)		380	(64.5)		
Partial vaccination^g^	0	(0.0)	3	(1.2)		22	(3.8)		
Full dose vaccination									
RV1 2 doses	3	(4.7)	41	(15.8)		121	(21.0)		
RV5 3 doses	0	(0.0)	38	(14.6)		53	(9.2)		

BW, birth weight; HC, hospital control; IQR, interquartile range; SD, standard deviation; TNC, test negative control.

^a^Chi-squared test or Wilcoxon’s rank-sum test was used as appropriate.

^b^Data were missing in 3 test-negative controls, and 2 hospital controls.

^c^Data were missing in 3 cases, 7 test-negative controls, and 2 hospital controls.

^d^Data were missing in 1 case, 4 test-negative controls, and 2 hospital controls.

^e^Data were missing in 8 cases, 6 test-negative controls, and 19 hospital controls.

^f^Data were missing in 3 test-negative controls and 6 hospital controls.

^g^Received one or two doses of RV5 or one dose of RV1.

Table 2 shows the basic characteristics and clinical symptoms of cases and test-negative controls. The proportion of subjects with severe symptoms was significantly higher in cases than in test-negative controls. There were no vaccinated patients among the severe cases.

**Table 2.** Clinical symptoms and treatment of cases and test-negative controls

Variables	Cases, *n* = 64		TNC, *n* = 260		*P*-value^a^ comparing Cases and TNC
	
	Rotavirus-positive		Rotavirus-negative		
Systemic symptoms before the medical examination					
Diarrhea, *n* (%)	60	(93.5)	224	(86.2)	0.10
Number of diarrheal stools, median [IQR]	4	[2.5–7.5]	3	[2.0–5.0]	<0.01
Vomiting, *n* (%)	48	(75.0)	119	(45.7)	<0.01
Number of vomiting episodes, median [IQR]	2.5	[2.0–4.5]	2	[1.0–4.0]	0.07
Fever, *n* (%)	45	(70.3)	108	(39.4)	<0.01
Max recorded fever, median [IQR]	38.5	[38.0–39.0]	38.4	[37.9–39.0]	0.83
Severity of disease^b^, *n* (%)					<0.01
Mild	30	(46.9)	219	(84.2)	
Moderate	21	(32.9)	32	(12.3)	
Severe	13	(20.3)	9	(3.5)	

HC, hospital control; IQR, interquartile range; SD, standard deviation; TNC, test negative control.

^a^Chi-squared test or Wilcoxon’s rank-sum test was used as appropriate.

^b^Severity of disease was assessed using the severity score (see the Methods section).

“Mild” corresponds to a total score of 1–4, “Moderate” corresponds to 5–6, and “Severe” corresponds to 7–9.

After adjustment for potential confounders, the estimated VEs against RVGE using test-negative controls and hospital controls were 84.9% (95% CI, 49.6–95.5%) and 86.6% (95% CI, 55.9–96.0%), respectively. There was no association between vaccination and non-rotavirus-related gastroenteritis (Table 3).

**Table 3.** Vaccine effectiveness against rotavirus gastroenteritis

	Cases		Controls		Crude OR (95% CI)	Adjusted OR (95% CI)	VE % (95% CI)
	
	*n*	(%)	*n*	(%)			
**Case-vs-test-negative control study**							
Unvaccinated	61	(95.3)	178	(68.5)	Reference	Reference	
Vaccinated (≥1 doses)	3	(4.7)	82	(31.5)	0.11 (0.03 to 0.35)	0.15^a^ (0.05 to 0.50)	84.9% (49.6 to 95.5)

**Case-vs-hospital control study**							
Unvaccinated	61	(95.3)	380	(64.5)	Reference	Reference	
Vaccinated (≥1 doses)	3	(4.7)	209	(35.5)	0.09 (0.03 to 0.29)	0.13^b^ (0.04 to 0.44)	86.6% (55.9 to 96.0)

**Rotavirus negative gastroenteritis^d^-vs-non-gastroenteritis^e^ study**							
Unvaccinated	178	(68.5)	380	(64.5)	Reference		
Vaccinated (≥1 doses)	82	(31.5)	209	(35.5)	0.84 (0.61 to 1.14)	0.95^c^ (0.69 to 1.32)	5.5% (−31.7 to 31.2)

CI, confidence interval; OR, odds ratio; VE, vaccine effectiveness.

^a^Adjusted for age in months, use of daycare and medical facility.

^b^Adjusted for age in months, use of daycare and medical facility.

^c^Adjusted for sex and medical facility.

^d^Test-negative control was reworded.

^e^Hospital control was reworded.

## DISCUSSION

In the present study, we evaluated rotavirus VE against RVGE using test-negative controls and hospital controls and compared VEs to assess whether or not test-negative controls are useful. Rotavirus vaccines were highly effective against RGVE, as documented via the VEs estimated using both hospital controls and test-negative controls (86.6% [95% CI, 55.9–96.0%] and 84.9% [95% CI, 49.6–95.5%], respectively). The VEs estimated using either type of control were almost equally high and were similar to those reported from previous clinical trials,^3^^,^^4^ confirming the effectiveness of rotavirus vaccines in the real-world setting in Japan.

It is difficult to evaluate VE estimated using a cohort design in Japan because there is no public vaccination registry corresponding to the Immunization Information System in the United States and no available data from surveillance for outpatient visits or hospitalization for RVGE. To date, to the best of our knowledge, there are only two studies that evaluated rotavirus vaccine effectiveness in Japan.^18^^,^^19^ One is our own previous case-population study in Saga Prefecture, which reported a 69.5% VE against RVGE and 88.8% VE against severe RVGE requiring hospitalization.^18^ That study was important for monitoring VE; it was the first on VE undertaken shortly after introduction of the rotavirus vaccines. However, a limitation of that study was that possible confounding factors were not adjusted for. The second study, the design of which was a case-versus-test-negative control study, was conducted at a single hospital in Akita Prefecture. The VE against severe RVGE requiring hospitalization was calculated as 70.4% in that study.^19^ The estimate of VE (86.6% [95% CI, 55.9–96.0]) in our present study was higher than previous studies in Japan, although the confidence interval overlaps those of previous studies, as well as those from other developed countries.^20^^–^^22^

As expected, comparing test-negative controls with hospital controls indicated that patient vaccination status was not associated with hospital visits due to rotavirus-negative acute gastroenteritis. Thus, there was no confounding bias, such as that which could have been caused by differences in health-related knowledge or practice to prevent gastroenteritis, between rotavirus vaccination and hospital visiting due to rotavirus-negative acute gastroenteritis. In addition, the VEs against RGVE estimated using the two types of controls overlapped substantially, indicating that bias from healthcare-seeking behaviors in case-versus-test-negative control study is similar to those in case-versus-hospital controls study. We therefore conclude that a test-negative control design is useful for determining the effectiveness of rotavirus vaccines.

The use of test-negative controls also has some other advantages relative to hospital controls, in addition to cancelling out bias due to differences between cases and controls regarding healthcare-seeking behaviors manifesting as hospital visits.^10^ The first is that using test-negative controls is more time-efficient and resource-saving, because cases and controls can be recruited in the same setting of acute gastroenteritis using the same immunochromatographic assay. One important point here is that the test should be applied on all patients of target age with symptoms of acute gastroenteritis. The second is that the case-versus-test-negative control design reduces possible confounding factors, because the latter are investigated before the results of the rotavirus test are known.^6^

The present study has some limitations. First, a test-negative control design would not reduce selection bias for receiving vaccination due to differences in healthcare-seeking behaviors. People with good healthcare-seeking behavior may be more likely to have their children vaccinated to try to avoid the chance of infection with rotavirus; if so, VE might be overestimated. However, it is more usual that healthcare is sought at the occurrence of symptoms, so a test-negative control design is considered useful. Second, the VE in our study was estimated for at least one vaccine dose versus no dose, which might result in lower VE estimates. Although we could not calculate the VE according to vaccine dose received, because no partial vaccination cases were included in this study, a previous report had indicated that VE for two doses was higher than for one dose only.^23^ Third, the type of vaccine, RV1 or RV5, could not be taken into account in the present study because none of the cases had received RV5 vaccine. However, VEs for RV1 and RV5 have been reported to be similar in other studies,^22^ but none of these were from Japan. Fourth, we could not evaluate VE against severe RVGE, because there were no severe cases in the vaccinated group, although severe RVGE may be a common outcome measure for rotavirus VE. Finally, false-positive cases might have occurred because immunochromatography might have detected vaccine antigens relatively shortly after vaccination. To reduce such misclassification, we did not include acute gastroenteritis patients who had been vaccinated within 14 days prior to presentation.

In conclusion, we have documented that rotavirus vaccines are highly effective in preventing RVGE among children <2 years of age in Japan. The values of VEs estimated using two different sets of controls were nearly identical and were similar to the VEs reported from other developed countries. Thus, these data indicate that a test-negative control design is a useful, efficient, and resource-saving technique for determining the effectiveness of rotavirus vaccines.
